# Prebiotics and Community Composition Influence Gas Production of the Human Gut Microbiota

**DOI:** 10.1128/mBio.00217-20

**Published:** 2020-09-08

**Authors:** Xiaoqian Yu, Thomas Gurry, Le Thanh Tu Nguyen, Hunter S. Richardson, Eric J. Alm

**Affiliations:** a Department of Biology, Massachusetts Institute of Technology, Cambridge, Massachusetts, USA; b Department of Biological Engineering, Massachusetts Institute of Technology, Cambridge, Massachusetts, USA; c Center for Microbiome Informatics and Therapeutics, Massachusetts Institute of Technology, Cambridge, Massachusetts, USA; d Pharmaceutical Biochemistry Group, School of Pharmaceutical Sciences, University of Geneva, Geneva, Switzerland; e The Broad Institute of MIT and Harvard, Cambridge, Massachusetts, USA; Nanyang Technological University

**Keywords:** functional heterogeneity, gut microbiome, intestinal gas, prebiotics

## Abstract

Prebiotics confer benefits to human health, often by promoting the growth of gut bacteria that produce metabolites valuable to the human body, such as short-chain fatty acids (SCFAs). While prebiotic selection has strongly focused on maximizing the production of SCFAs, less attention has been paid to gases, a by-product of SCFA production that also has physiological effects on the human body. Here, we investigate how the content and volume of gas production by human gut microbiota are affected by the chemical composition of the prebiotic and the community composition of the microbiota. We first constructed a linear system model based on mass and electron balance and compared the theoretical product ranges of two prebiotics, inulin and pectin. Modeling shows that pectin is more restricted in product space, with less potential for H_2_ but more potential for CO_2_ production. An *ex vivo* experimental system showed pectin degradation produced significantly less H_2_ than inulin, but CO_2_ production fell outside the theoretical product range, suggesting fermentation of fecal debris. Microbial community composition also impacted results: methane production was dependent on the presence of *Methanobacteria*, while interindividual differences in H_2_ production during inulin degradation were driven by a *Lachnospiraceae* taxon. Overall, these results suggest that both the chemistry of the prebiotic and the composition of the microbiota are relevant to gas production. Metabolic processes that are relatively prevalent in the microbiome, such as H_2_ production, will depend more on substrate, while rare metabolisms such as methanogenesis depend more strongly on microbiome composition.

## Abstract (precis)

Prebiotic fermentation in the gut often leads to the coproduction of short-chain fatty acids (SCFAs) and gases. While excess gas production can be a potential problem for those with functional gut disorders, gas production is rarely considered during prebiotic design. In this study, we combined the use of theoretical models and an *ex vivo* experimental platform to illustrate that both the chemical composition of the prebiotic and the community composition of the human gut microbiota can affect the volume and content of gas production during prebiotic fermentation. Specifically, more prevalent metabolic processes such as hydrogen production were strongly affected by the oxidation state of the probiotic, while rare metabolisms such as methane production were less affected by the chemical nature of the substrate and entirely dependent on the presence of *Methanobacteria* in the microbiota.

## INTRODUCTION

The gut microbiota plays an important role in human nutrition and health, leading to increasing interest in modulation of the gut microbiome via dietary interventions for improving human health (1–3). Compounds that can be selectively metabolized by microbes in the gut resulting in beneficial effects on the host are defined as prebiotics (4). While some phenolic compounds and fatty acids are suspected to have prebiotic activities, most known prebiotics are dietary carbohydrates that are neither digested nor absorbed in the human small intestine and are thus capable of reaching the colon and promoting the growth of selective beneficial bacteria (4, 5). These bacteria, in turn, can prevent the colonization of pathogens or produce metabolites that are beneficial for the human body, most notably short-chain fatty acids (SCFAs) such as acetate, propionate, and butyrate. These SCFAs not only contribute directly to host energy metabolism but have a number of positive effects on host physiology. Butyrate is the major energy source for colonocytes and enterocytes (6) and can also activate gluconeogenesis and modulate inflammatory responses and cytokine levels via G protein‐coupled receptors or histone deacetylases (7). Similarly, acetate and propionate are involved in the regulation of host immune or metabolic systems (7, 8). Thus, selection for prebiotics has largely focused on those that allow the proliferation of bacteria that maximize production of SCFAs (5, 9, 10).

SCFA fermentation from carbohydrates by the gut microbiota is often coupled with the production of gases. Production of H_2_ is often necessary for the cycling of NAD^+^/NADH during fermentation, and CO_2_ is released whenever decarboxylation occurs (11). H_2_ can be further utilized by methanogens and sulfate reducers for the production of CH_4_ and H_2_S (12). Most intestinal gas is absorbed into the bloodstream and removed via the lungs (13), but it can still have physiological effects on the human body. The volume of gas production can affect the distension of the colonic wall and in turn affect the speed of material transition through the colon (14). Methane production can result in slowed intestinal transit and reduced serotonin levels in the gastrointestinal tract, potentially impacting constipation-predominant irritable bowel syndrome (IBS-C) and chronic constipation (15). Therefore, gas production may be an important factor to consider in the selection of prebiotics, especially since bloating is a major symptom for many functional gut disorders such as IBS (16).

Many prebiotics are already known to impact fermentation products. For example, short-chain fructooligosaccharides (FOS) and inulin are some of the most extensively documented prebiotics, because they promote the growth of bifidobacteria and increase SCFA production (4, 5). However, the low-FODMAP (fermentable oligosaccharide, disaccharide, monosaccharide, and polyol) diet has been shown to improve IBS symptoms in some patients, because foods containing FOS and inulin can increase luminal distension and gas production (17, 18). Thus, it may be valuable to identify prebiotics that maximize SCFA and minimize gas production or minimize the production of specific gases. However, few studies that consider the efficacy of prebiotics simultaneously take gas and SCFA production into account, and systematic investigations on factors that affect gas production in prebiotic fermentation are lacking.

In this study, we investigated whether the chemical composition of the prebiotic and heterogeneity in the compositions of gut microbiota can affect the content and volume of gas production during prebiotic fermentation. We compared the fermentation products of two common prebiotics, inulin and pectin, both theoretically via linear system modeling and experimentally via an *ex vivo* framework that measures gas and SCFA production of stool microbiota responding to fiber addition (19). We find that inulin, a more reduced carbohydrate, produces more H_2_ than pectin, but the amount of H_2_ production is strongly associated with a *Lachnospiraceae* amplicon sequencing variant (ASV). Inulin also yielded greater amounts of the more reduced SCFA butyrate and less acetate. Methane production is, however, less affected by the chemical nature of the substrate, being entirely dependent on the level of *Methanobacteria* in the microbiota. Overall, these results suggest that the production of different gases upon prebiotic fermentation by gut microbiota are differentially affected by the chemical nature of the prebiotic and microbiome compositions.

## RESULTS

### Modeling community production with mass and electron balance.

**FIG 1:**
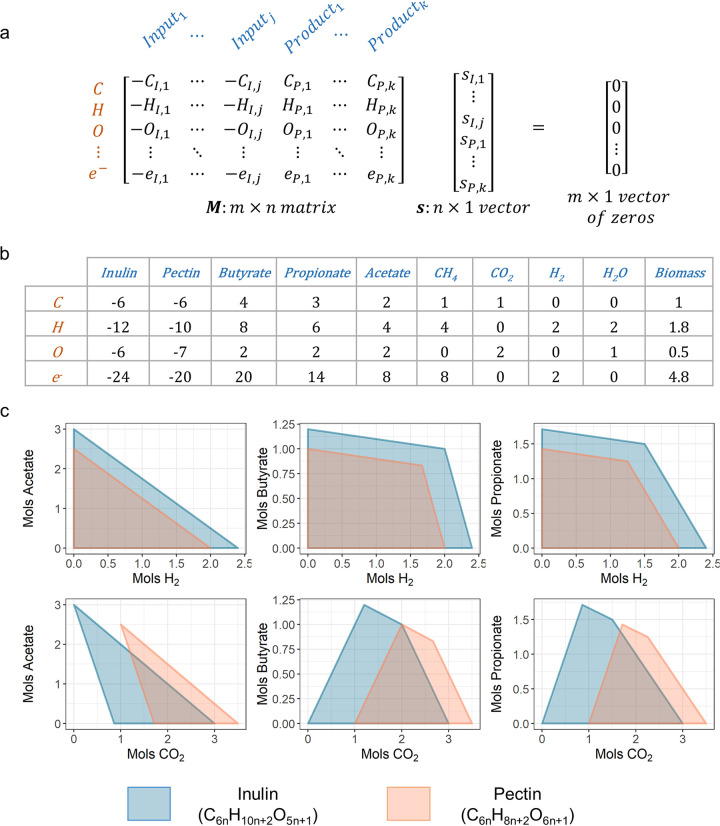
Modeling community production with mass and electron balance. (a) Detailed representation of the theoretical model **Ms = 0**, where **M** is a matrix with **n** chemicals (j inputs and k products). In **M**, inputs are represented in negative numbers, while outputs are represented in positive numbers. Each row in **M** represents an element (or electrons) that needs to be balanced. (b) The specific **M** matrix corresponding to our system of interest, fermentation of two different fibers, inulin and pectin. (c) Set of selected 2D projections of the feasible product space predicted from our theoretical model for the fermentation of 1 mol inulin or 1 mol pectin. See Fig. S1 in the supplemental material for the full set of 2D projections for all fermentation products.

To explore the general effect of prebiotic chemical composition on fermentation product formation, we established a linear system model that allowed us to determine the theoretical range of product output considering mass and electron balance. Considering a system of **n** chemicals as possible inputs and outputs, made up of a total of **m** chemical elements, we defined a matrix **M** in which the rows represent different elements and columns represent different chemicals; the elemental composition of a chemical is thus a column in **M**. The total number of valence electrons in the chemical is also counted as an “element” and consists of a separate row in **M**. Thus, any reaction that satisfies both mass and electron balance is an n-dimensional vector **s**, whose elements are the stoichiometric coefficients of the chemicals in **M** and satisfy **Ms = 0** (Fig. 1a). By definition, **s** must be within the null space of **M**. The feasible product space of the biological system, represented by the elements in **s** that are coefficients of the possible products, is thus a convex cone defined by the linear combinations of the basis vector of null(**M**). Since our model did not account for the thermodynamic constraints on the metabolic fluxes within the system, it represented an upper limit of the feasible product space.

10.1128/mBio.00217-20.1 FIG S1 2D projections of the feasible product space for all fermentation products. The full set of 2D projections of the feasible product space predicted from our theoretical model for the fermentation of 1 mol inulin or 1 mol pectin. Units on all axes are mol product/mol fiber. Download FIG S1, JPG file, 1.0 MB. Copyright © 2020 Yu et al. 2020 Yu et al. This content is distributed under the terms of the Creative Commons Attribution 4.0 International license.

10.1128/mBio.00217-20.2 FIG S2a 2D projections of the feasible product space for all fermentation products assuming constant biomass yield. The full set of 2D projections for the feasible product space predicted from our theoretical model for the fermentation of 1 mol inulin or 1 mol pectin, when assuming that 15% of the C in fiber is eventually converted into biomass. Units on all axes are mol product/mol fiber. Download FIG S2a, TIF file, 2.9 MB. Copyright © 2020 Yu et al. 2020 Yu et al. This content is distributed under the terms of the Creative Commons Attribution 4.0 International license.

10.1128/mBio.00217-20.3 FIG S2b 2D projections of the feasible product space for all fermentation products assuming constant biomass yield. Comparison of the 2D projections for the feasible product space predicted from our theoretical model for 1 mol of inulin with no restriction on biomass yield (light blue) and assuming a 15% biomass yield during the fermentation process (dark blue). Download FIG S2b, TIF file, 3.0 MB. Copyright © 2020 Yu et al. 2020 Yu et al. This content is distributed under the terms of the Creative Commons Attribution 4.0 International license.

10.1128/mBio.00217-20.4 FIG S2c 2D projections of the feasible product space for all fermentation products assuming constant biomass yield. Comparison of the 2D projections for the feasible product space predicted from our theoretical model for 1 mol of pectin with no restriction on biomass yield (light coral) and assuming a 15% biomass yield during the fermentation process (coral). Download FIG S2c, TIF file, 2.9 MB. Copyright © 2020 Yu et al. 2020 Yu et al. This content is distributed under the terms of the Creative Commons Attribution 4.0 International license.

### Feasible product space of pectin fermentation is more limited than that of inulin.

We applied our model to compare the feasible product space for the fermentation of 1 mol of inulin (C_6n_H_10n+2_O_5n+1_) to that of 1 mol of pectin (C_6n_H_8n+2_O_6n+1_) in a closed system. Since we were modeling product output from carbohydrate input, we only included C, H, O, and valence electrons as rows in our matrix **M**. For products (columns) in **M**, we included the three most abundant SCFAs in the gut (acetate, propionate, and butyrate) and the three major components of intestinal gas (H_2_, CH_4_, and CO_2_) as well as water and biomass (represented by CH_1.8_O_0.5_N_0.2_, the mean chemical formula for microbial biomass [20]) (Fig. 1). All product concentrations were restricted to be nonnegative to simulate a closed system (i.e., product formation is solely from fiber input). Our model showed that in a closed system, the product space of pectin was more restricted than that of inulin (see Fig. S1 in the supplemental material); in particular, inulin had more potential for H_2_ production, while pectin had more potential for the production of CO_2_ (Fig. 1c). Model predictions were conserved even if further constraints were placed on the system, i.e., 15% of C in the fiber is converted into biomass as in a typical carbohydrate fermentation (Fig. S2a, S2b, and S2c) (21).

### Pectin degradation takes up reducing agents from the environment.

**FIG 2:**
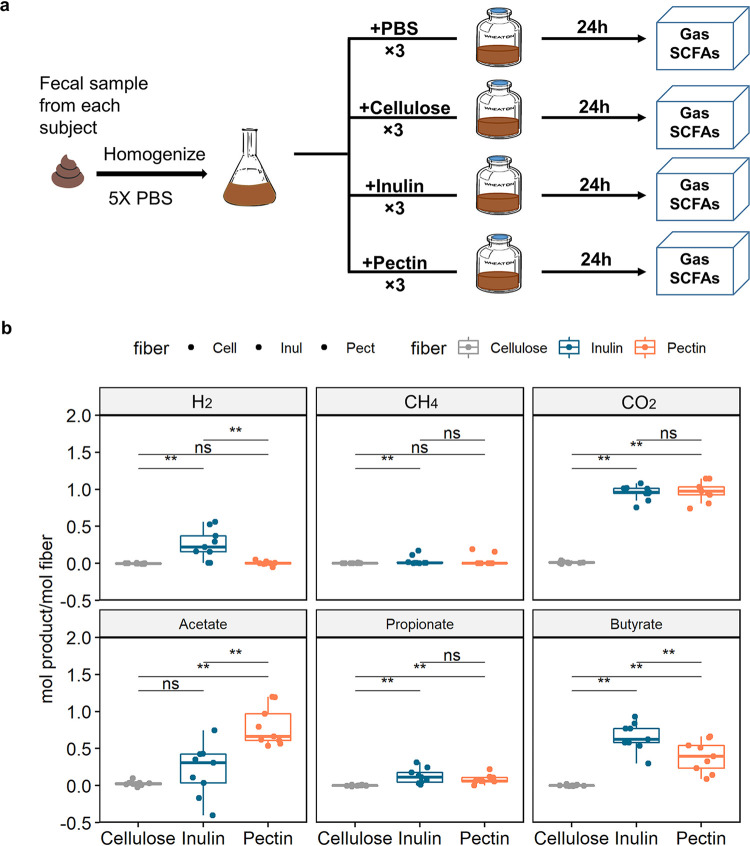
Inulin fermentation produces more H_2_ and less acetate than pectin in an *ex vivo* system. (a) Experimental scheme for studying fiber fermentation products in an *ex vivo* system. (b) Major product concentrations in the *ex vivo* system after 24 h measured as moles product production per mole of fiber. **, *P* < 0.01 for paired Kruskal-Wallis test; ns, not significant.

We next asked if our theoretical predictions could be experimentally validated using an *ex vivo* framework in which we measured the response of stool microbiota to fiber addition. First, stools from 9 healthy human subjects were each homogenized with phosphate-buffered saline (PBS) under anaerobic conditions to create fecal slurries. The slurries were then incubated in serum bottles at 37°C starting with 100% N_2_ in the headspace, with inulin, pectin, cellulose, or no additional fiber input (Fig. 2a). Since our preliminary testing showed that degradation of soluble fibers in this system was almost entirely complete within 24 h (19), we used the gas and SCFA concentrations at 24 h as the experimental product concentrations for comparison to those predicted from our theoretical models. Because the fecal slurry itself contained a certain amount of residue material from food digestion in the human body, even samples that did not receive additional fiber produced gas and SCFAs; the fermentation products of a certain fiber in a sample were thus determined as the difference between product concentrations measured in a sample which received additional fiber and those in a sample which did not. We found the gas and SCFA concentrations of samples with cellulose was not significantly different from the control samples, likely because cellulose was not soluble in water and precipitated as a thick white layer at the bottom of the serum bottles. We thus focused our analyses on samples with soluble fiber input, i.e., inulin and pectin.

**FIG 3:**
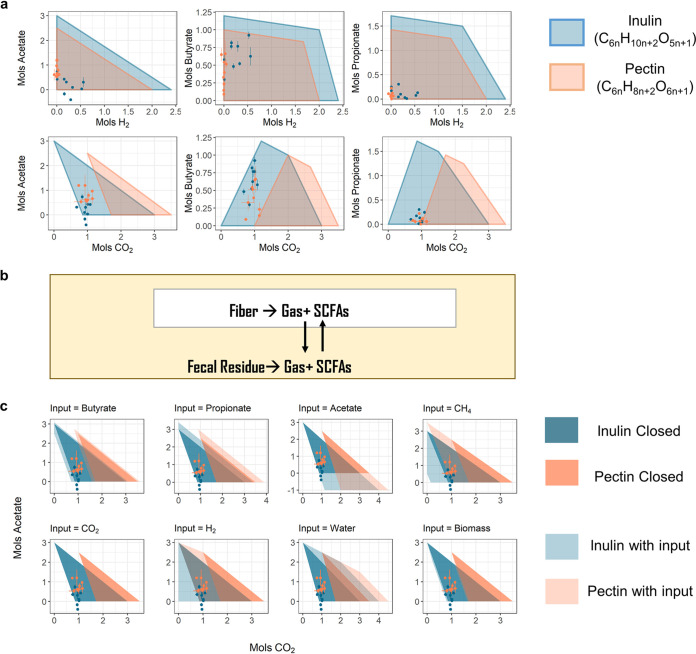
Pectin degradation requires the uptake of reducing agents. (a) Comparison of product measurements in the *ex vivo* system to theoretically predicted feasible product ranges. (b) Illustration of the two subsystems within the serum bottle and their material exchanges. (c) Comparison of product measurements in the *ex vivo* system to theoretically predicted feasible product ranges in a closed system and when allowing different inputs. Error bars in panels a and c represent standard deviations from biological replicates.

Focusing on gas production in the *ex vivo* systems, we found that the amount of H_2_ produced by pectin fermentation was significantly lower than that of inulin (Fig. 2b) (Kruskal-Wallis test, paired, *P* = 0.004), and the total amount of gas production was also lower (Kruskal-Wallis test, paired, *P* = 0.07). However, we did not observe a larger amount of CO_2_ production in pectin fermentation than in inulin fermentation as theoretically predicted; in fact, the measured CO_2_ productions from pectin fermentation did not fall within the previous theoretically predicted range (Fig. 3a). This was also the case for acetate production in some samples that fermented inulin. Since our model only considers the most basic laws of chemistry and represents the maximum possible theoretical product range for a closed system, we hypothesized that the experimental violation of the results from the theoretical model was due to assuming that our experimental system was closed. Indeed, despite the serum bottle being a closed system with no material exchange with the environment outside the bottle, fermentation of the additional fiber should be seen as a subsystem that can exchange products with the other subsystem in the bottle that ferments residue material in the fecal slurry (Fig. 3b).

We thus investigated what input the “fiber subsystem” would need from the “residue subsystem” for the measured CO_2_ to fall within the feasible product range determined by the theoretical model. Since, on average, the samples that did not receive additional fiber produced approximately one-third as much gas and SCFAs as those that did (see Fig. S4), we limited the input from the residue subsystem to the equivalent amount of product that can be produced by 1/3 mol of inulin or pectin. Allowing one input at a time, we found that only when H_2_ or CH_4_ was used as input would the measured CO_2_ fall within the predicted range (Fig. 3c). Although we did not observe net uptake of either H_2_ or CH_4_ in our experimental data, but because both H_2_ and CH_4_ are chemicals with reducing power, there was likely influx of other reducing substrates not presented in our model from the “residue subsystem” to the “fiber subsystem.” Thus, in our *ex vivo* system, pectin degradation not only had a lower net production of H_2_ than that of inulin but also took up reducing agents from the surrounding environment. We thus speculate that when pectin is degraded in the human gut, it is also taking up reducing agents—a process for which consequences are unclear and possibly worth further investigation.

### H_2_, acetate, and butyrate distinguish the product profile of inulin and pectin degradation.

**FIG 4:**
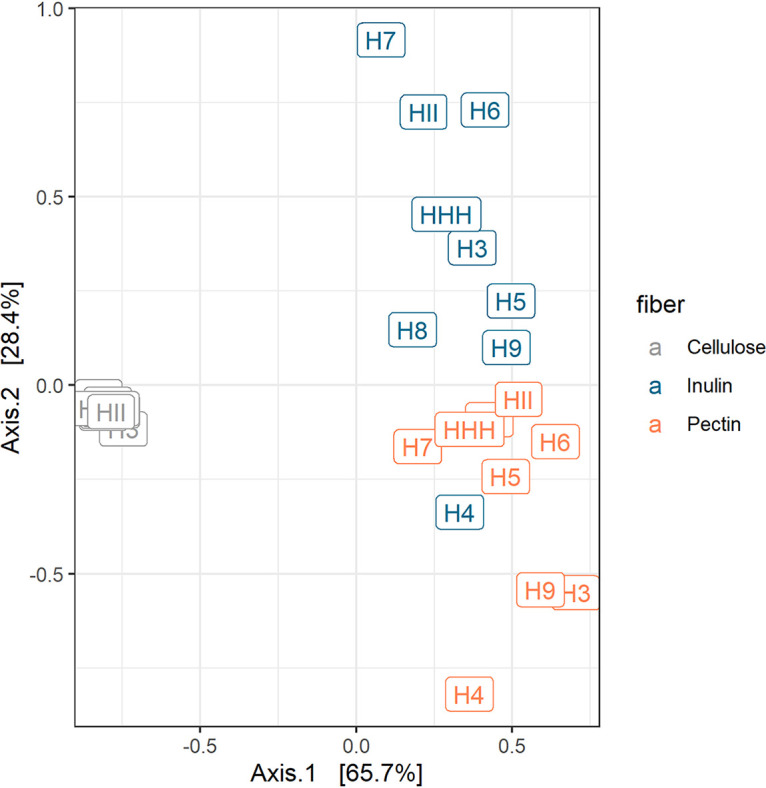
Principal-coordinates analysis (PCoA) of the Euclidian distance matrix of the fiber fermentation products from the *ex vivo* system. Each point corresponds to a community colored according to the fiber added to the system. Each point is labeled by the source (human subject [H]) from which the stool sample was collected.

We next asked if the overall product profiles of inulin degradation and pectin degradation can be distinguished from each other and whether changing the fiber or microbial community contributed more to the variation in product profiles. We found that product profiles primarily clustered by fiber and secondarily by human subjects (Fig. 4) (permutational multivariate analysis of variance [PERMANOVA], *R*^2^_(fiber)_ = 0.79, *P*_(fiber)_ = 0.001; *R*^2^_(subjects)_ = 0.12, *P*_(subjects)_ = 0.02). Given that inulin fermentation generated significantly more H_2_ and butyrate but less acetate that that of pectin (Fig. 2b), we hypothesized these are the three major products that would allow the product profiles of inulin and pectin fermentations to be distinguished. Indeed, the top products found to separate the inulin and pectin samples in the PERMANOVA were acetate, butyrate, and H_2_. Importantly, the coefficients for the more reduced products, H_2_ and butyrate, were in the opposite direction of acetate (see Fig. S3). The more oxidized substrate, pectin, produced more of the most oxidized SCFA, acetate, while inulin produced more H_2_ and the most reduced SCFA, butyrate (Fig. 2b).

10.1128/mBio.00217-20.5 FIG S3 Coefficients of PERMANOVAs of fiber fermentation products separating the inulin and pectin samples. Download FIG S3, TIF file, 0.2 MB. Copyright © 2020 Yu et al. 2020 Yu et al. This content is distributed under the terms of the Creative Commons Attribution 4.0 International license.

10.1128/mBio.00217-20.6 FIG S4 Ratios of products in control versus treatment groups. The gas or SCFAs in each sample were summed together by their carbon (C), hydrogen (H), or oxygen (O) content. Only the samples with inulin or pectin addition were counted in the treatment group. Download FIG S4, TIF file, 0.2 MB. Copyright © 2020 Yu et al. 2020 Yu et al. This content is distributed under the terms of the Creative Commons Attribution 4.0 International license.

### Relative effects of microbiome and substrate chemistry on gas production differ among gases.

**FIG 5:**
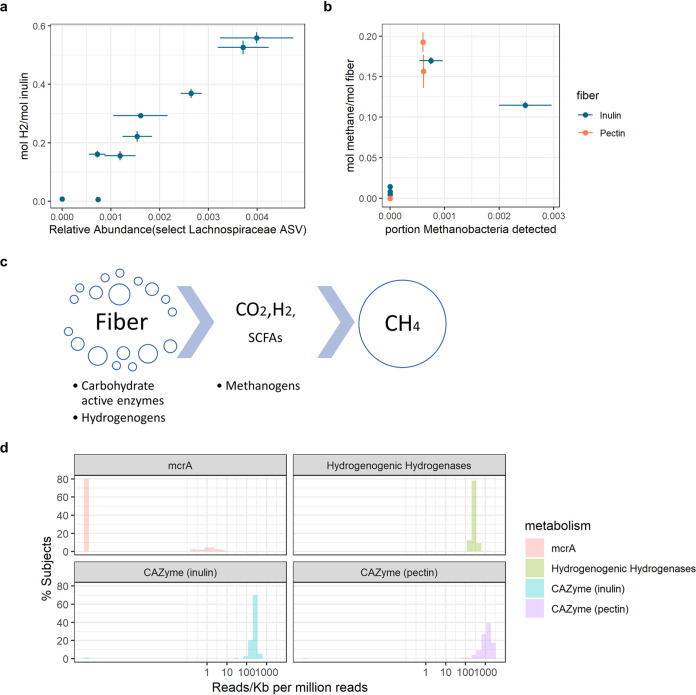
Different gases are differentially influenced by substrate chemistry and gut microbiome composition. (a) Relationship between the observed amount of H_2_ production per mole of inulin in the *ex vivo* system and the relative abundance of the *Lachnospiraceae* ASV selected by the Lasso regression. Error bars represent standard deviations from biological replicates (*n* = 3). (b) Relationship between the production of methane per mole of fiber in the *ex vivo* system and the relative abundance of *Methanobacteria* in the samples. Error bars represent standard deviations from biological replicates (*n* = 3). (c) Schematic of fiber degradation and production of gas and SCFAs. (d) Distributions of the abundances of the methanogenesis marker gene *mcrA*, hydrogenic hydrogenases, and CAZymes in the metagenomes of 160 different people in the HMP data set. All gene counts were increased by 10^−5^ so that the log-scaled *x* axis could accommodate samples with zero hits.

We further explored if there were signatures within the microbiomes that promoted the production of gases. Since levels of H_2_ production were generally low for pectin fermentation, we investigated if there were specific ASVs associated with net H_2_ production during inulin fermentation. Selecting for these ASVs via Lasso regression identified a *Lachnospiraceae* amplicon sequencing variant (ASV) positively associated with net H_2_ production (Fig. 5a) (Pearson’s *r* = 0.97, *P* = 1.14 × 10^−5^) (see Fig. S5). Since net H_2_ production in the gut is the difference between the total production of H_2_ and the total consumption of H_2_ (12), and *Lachnospiraceae* can be either hydrogen producers or consumers (22, 23), the positive association of the *Lachnospiraceae* ASV with H_2_ production in the human gut may indicate that net H_2_ production is more dependent on H_2_ production than on consumption. Consistent with this hypothesis, H_2_ consumption abilities of gut microbiota may be more consistent between different people than H_2_ production because of the higher diversity of H_2_ consumption pathways (methanogenesis, reductive acetogenesis, and sulfate reduction) than those for production. It was, however, observed that net H_2_ production was lowest in the samples that produced methane, probably because the amount of sulfate in the *ex vivo* system is not enough to for the most energetically favorable H_2_ consumption pathway, sulfate reduction, to consume all the H_2_ produced (see Text S1), and methanogenesis is more energetically favorable than reductive acetogenesis (24).

10.1128/mBio.00217-20.7 FIG S5 Evaluation of Lasso regression for H_2_ production in samples containing inulin. Relationship between the predicted value of H_2_ production per mol of inulin based on a linear model with a *Lachnospiraceae* ASV as the independent variable, and the observed amount of H_2_ production per mol of inulin in *ex vivo* system. Dotted line represents a linear fit of the relationship, with grey areas around the line representing the standard error of the fit. Download FIG S5, TIF file, 0.1 MB. Copyright © 2020 Yu et al. 2020 Yu et al. This content is distributed under the terms of the Creative Commons Attribution 4.0 International license.

10.1128/mBio.00217-20.9 TEXT S1 Estimation of sulfate content in an *ex vivo* system. Download Text S1, DOCX file, 0.01 MB. Copyright © 2020 Yu et al. 2020 Yu et al. This content is distributed under the terms of the Creative Commons Attribution 4.0 International license.

In contrast to H_2_, whose production was strongly affected by substrate, we found that methane production was solely dependent on whether there were detectable levels of *Methanobacteria* in the microbiota (Fig. 5b). Given that methane is a downstream product of H_2_ (Fig. 5c), we asked why methane production was not affected by substrate chemistry but H_2_ production was. We hypothesized that this is because there are generally large amounts of bacteria in the human gut that contain carbohydrate-active enzymes and hydrogenases that allow fiber breakdown and hydrogen production; thus, the amounts of these enzymes are not a limiting factor, allowing hydrogen production to be instead dependent on substrate stoichiometry. Meanwhile, a large portion of the human population does not harbor sufficient numbers of methanogens, making them the limiting factor for methane production; however, when the number of methanogens is sufficient, they are not limited by the amount of H_2_ production because methanogens are stronger competitors for H_2_ than reductive acetogens. To test this hypothesis, we surveyed the abundances of carbohydrate-active enzymes (CAZymes) for inulin and pectin as well as hydrogenogenic hydrogenases and methyl coenzyme M (methyl-CoM) reductases (*mcrA*, marker gene for methanogens) in the metagenomes of 160 randomly selected healthy human subjects from the human microbiome project (HMP). While only approximately 20% of subjects had detectable levels of methanogens, nearly all subjects harbored CAZymes for inulin and pectin degradation as well as hydrogenogenic hydrogenases (Fig. 5d). The percentage of subjects (20%) with detectable methanogens in the HMP data is in accordance with our results: 2 of the 9 subjects were methane producers in our *ex vivo* experiment. Thus, overall, the production of more-“general” metabolites such as H_2_ is more likely to be affected by the chemical composition of the prebiotic, while more-“rare” metabolites such as methane are more likely to be limited by the organisms that produce it.

## DISCUSSION

In this study, we used a combination of theoretical models and an *ex vivo* experimental framework to examine how the chemistry of prebiotics and the composition of the gut microbiota influence gas production during prebiotic fermentation by gut microbiota. Specifically selecting two different common prebiotics (inulin and pectin) with different levels of oxidation, we find that metabolites that can be produced by more organisms in the human gut, such as H_2_, are more affected by the chemical composition of prebiotics than metabolites that are produced by less common organisms in the gut, such as methane. Overall, these results suggest that both the chemical nature of the prebiotic and the individual’s gut microbiome needs to be considered when administering prebiotics to individuals.

Our data also reveal that there may be general trade-offs in the production of SCFAs versus that of gas. For example, while inulin fermentation leads to more production of the more reduced SCFA butyrate, it also leads to more production of the reducing agent H_2_, in turn, increasing overall gas production. However, which is more preferable for the subject—more production of butyrate, less production of overall gas, or just less production of H_2_—is often unknown and specific to the individual subject. This can be further complicated if interindividual differences in H_2_ and SCFA production are considered: not every individual produces more butyrate when fermenting inulin. Similarly, for pectin, we were able to infer by comparing the experimental data to the theoretical product range that pectin degradation requires uptake of reducing agents from the surrounding environment. Again, what effect this has on the host is unknown: would the uptake of these reducing agents lead to the generation of more reactive oxygen species that can directly attack cells in the gut epithelial barrier, interfere with iron uptake, or initiate lipid peroxidation processes (25)? Would this be costlier to the host than generating more H_2_? More importantly, we also lack a way to evaluate if the scale of the differences is large enough for them to count as a factor in prebiotic selection.

These problems emphasize that the effect of prebiotics on gut and human health must be looked at from both individual and systems perspectives. Often, a compound is deemed as a prebiotic because it can increase the growth of known beneficial microbes such as bifidobacteria and lactobacilli or promote the production of target metabolites. However, the full diversity of a mixed-culture environment such as the human gut must be considered when selecting for prebiotics: it is very hard to only selectively grow organisms that produce one or a few metabolites of interest, and the effect of any by-products must be considered. Interindividual differences in product formation due to heterogeneity in gut microbiota composition, as well as responses to the metabolites produced, must also be considered. Our use of theoretical modeling and the *ex vivo* experimental system to explore gas production and its relationship to SCFA production is just a beginning: these are relatively inexpensive and simple methods to shine light on important points that should be considered in prebiotic design.

The *ex vivo* experimental system can be seen as where techniques originating from many fields come together in a compromised way. Compared to the breath test often used in clinical practice for diagnosis of lactose intolerance or small intestinal bacterial overgrowth (26), it fails to measure gas production *in situ*; also, compared to cultures with known bacterial content and medium composition, it is more challenging to obtain measurements that can directly match the results of theoretical models. The fact that some of our experimental measurements do not fall within the predicted theoretical range reflects both the strength and weaknesses of our system: while our system is not perfect for testing hypotheses, it is a good system for pointing at possible directions and generating hypotheses that could be further tested. An important future improvement to the system would be better separation of the gut microbial community with the fecal matter from which it originates so that the experimental system truly mimics the “closed system” as described in the theoretical model. Specifically, a known reductive substrate could be added to the system to quantitatively measure the uptake of reducing agents during the degradation of different fibers.

Overall, our study is a first step toward developing a system where the unique microbiome composition of each individual can be measured simultaneously with its fermentation products for different kinds of fibers. In the future, a more systematic evaluation on what important factors other than the formation of beneficial metabolites should be considered in prebiotic design is needed.

## MATERIALS AND METHODS

### Experimental model and participant details.

Nine healthy human volunteers were enrolled into the study under the supervision of the MIT Committee on the Use of Humans as Experimental Subjects (COUHES), who approved the study under protocol number 1510271631. All participants provided written informed consent, and the study was conducted in accordance with the relevant guidelines and regulations. To be included, participants had to be between 18 and 70 years of age, have a body mass index (BMI) between 18 and 30, and not have a history of inflammatory bowel diseases/syndrome, type-2 diabetes, kidney diseases, intestinal obstruction, or colorectal cancer. They were also not currently pregnant or breastfeeding and had not received antibiotic treatment in the 6 months leading up to the study. Enrollment occurred between June 2017 and Oct 2017. The study group included 4 female and 5 male individuals, all between 25 and 40 years of age.

### Linear system model for modeling community production.

The product space for the system **Ms = 0** is a polytope defined by linear combinations of the basis vectors of null(M), i.e., **Bx = s**. Constraints on the product space (i.e., for the closed system, all elements in s corresponding to products are nonnegative) were used to find the vertices of the polytope of **x** by converting the half-space representation (the intersection of half spaces, represented by **Bx = s**) into vertex representation (set of extreme points of the polytope). Vertices of the polytope of **s** were calculated by multiplying **B** with the vertices of polytope **x**. The vertices for **s** were used to draw two-dimensional (2D) hulls for pairs of products to visualize the product polytope, as in Fig. 1c and 3a and c. When product input was allowed for the system, the constraint on the element in **s** corresponding to the input product was relaxed to be larger than the negative of the equivalent amount of product that can be produced by 1/3 mol of inulin or pectin (on average, control samples produced approximately one-third as much SCFA and/or gas than samples with inulin or pectin treatment) (see Fig. S4 in the supplemental material).

The basis set of vectors for the null space of matrix M was calculated from the QR-decomposition of the matrix using the R package “pracma” (27). The conversions of half-space representation to vertex representation of polytopes were performed using the R package “rcdd” (28).

### Setup of *ex vivo* system.

The setup of the *ex vivo* system was the same as that by Gurry et al. (19), with some adaptation for gas measurements. Briefly, fresh stool samples were collected and homogenized with reduced PBS containing 0.1% l-cysteine at a ratio of 1 g/5 ml. Fiber was spiked in to the homogenates from stock solutions such that the final concentrations of fibers in the samples were as follows: control (no fiber), 10 g/liter inulin, 5 g/liter pectin, or 20 g/liter cellulose. The concentration of the fibers spiked in were in accordance with the highest daily amount of the certain fiber that a healthy subject could ingest without experiencing discomfort determined in a previous *in vivo* dietary study (29) as well as with the relative solubility of the fibers (more total input if the fiber was less soluble). For each participant, 2 ml of the final fecal slurry of each condition was added in triplicates to 60-ml glass serum bottles (Supelco, Bellefonte, PA). The serum bottles containing the samples were transferred to a vinyl anaerobic chamber filled with 100% N_2_, with no detectable amounts of CO_2_ and H_2_, and sealed in the chamber using magnetic crimp seals with polytetrafluoroethylene (PTFE)-silicone septa (Supelco, Bellefonte, PA). A total of 12 bottles per participant were incubated at 37°C for 24 h with no shaking.

### Gas and SCFA measurements.

Concentrations of headspace gases were determined using gas chromatography. We used a Shimadzu GC-2014 gas chromatograph (GC) configured with a packed column (Carboxen-1000, 5 ft by 1/8 in. [Supelco, Bellefonte, PA]) held at 140°C, argon carrier gas, and thermal conductivity (TCD) and methanizer-flame ionization (FID) detectors. At the end of the 24-h incubation period, subsamples of the headspace (0.20 cm^3^ at the laboratory temperature, ca. 23°C) from each serum bottle were taken via a gas-tight syringe and injected into the column. Gas concentrations were determined by comparing the partial pressures of samples with standards of known concentrations. The accuracy of the analyses, evaluated from standards, was ±5%. Measurements of H_2_ were taken using the TCD while measurements of CH_4_ and CO_2_ were taken with the FID.

SCFA measurements were made from taking 1 ml of fecal slurry from each serum bottle immediately after the GC measurements were taken and freezing the fecal slurry at −80°C until time of measurement. SCFA measurements were made on an Agilent 7890B system with an FID at the Harvard Digestive Disease Core (Agilent Technologies, Santa Clara, CA). Detailed procedures for SCFA measurements are the same as those described by Gurry et al. (19). Although the amounts of 10 volatile acids (including acetic, propionic, isobutyric, butyric, isovaleric, valeric, isocaproic, caproic, and heptanoic acids) were reported, all but the acetic, butyric, and propionic acids were in trace amounts, and we only used these three SCFAs for our models.

### Machine learning and statistics.

The principlal-coordinate analyses of the *ex vivo* fermentation products were performed with the R package “ape” using Euclidian distance matrices (30). The alpha and beta diversities of the microbial community compositions were calculated with the R package “vegan.” PERMANOVAs were performed with the function adonis in the R package vegan (31), with 999 permutations. The Lasso regression was performed with the R package “glmnet” (32), and cross-validation was performed with a leave-one-out approach.

### DNA extraction, library preparation, and sequencing.

The Mo Bio PowerSoil-htp 96 kit (now Qiagen catalog number 12955-4), with minor modifications, was used to extract the DNA from all fecal samples. For all samples, 250 μl of the fecal slurry was used with the Mo Bio High Throughput PowerSoil bead plate (12955-4 BP). 16S rRNA gene amplicon libraries (V4 hypervariable region, U515 to E786) using a two-step PCR approach were prepared according to the method described by Preheim et al. (33). Samples were sequenced on an Illumina MiSeq (paired-end [PE] 150 + 150) at the Broad Walk-Up Sequencing platform (Broad Institute, Cambridge, MA). The average sequencing depth of the samples was 53,046 reads/sample.

### 16S rRNA amplicon data analysis.

All 16S rRNA amplicon libraries were processed according to a custom pipeline, where cutadapt (34) was used for primer trimming, QIIME 1.9 (35) was used for demultiplexing, and DADA2 (36) was used to infer amplicon sequence variants (ASVs). Default settings were used except that only the forward reads were used due to issues with merging reads, and the forward reads were truncated to 110 bp. Taxonomy for the sequence variants was assigned using the RDP database (37). The alpha and beta diversities of all communities are shown in Fig. S6a and b in the supplemental material.

10.1128/mBio.00217-20.8 FIG S6 Alpha and beta diversities of *ex vivo* microbial communities. (a) Box plots for the observed number of ASVs (left) and Shannon diversity (right) in each *ex vivo* microbial community. Top, middle, and lower hinge represent 75%, 50%, and 25% quantiles of the data, respectively. (b) PCoA plot of the Bray-Curtis dissimilarity of all *ex vivo* microbial communities. Colors represent different human subjects. Download FIG S6, TIF file, 0.5 MB. Copyright © 2020 Yu et al. 2020 Yu et al. This content is distributed under the terms of the Creative Commons Attribution 4.0 International license.

### Metagenome analysis.

We downloaded 160 randomly selected metagenomes from the human stool microbial communities of the Human Microbiome Project (National Institutes of Health, USA). Each metagenome was rarefactioned to 20 million reads (forward plus reverse) using seqtk seeded with the parameter –s100. The rarefactioned metagenomes were screened in DIAMOND (maximum number of high scoring pairs [HSPs] per subject sequence to save for each query = 1, blastx) against hydrogenogenic hydrogenases retrieved from the HydDB database (38), *mcrA* genes retrieved from the PhyMet database (39), and CAZymes from the dbCAN database (40). Results were then filtered (length of amino acid, >25 residues; percent identical matches, >65% [*mcrA* and hydrogenases] or >35% [CAZymes]). Reads were eventually normalized to reads per kilobase million using the formula $\frac{\text{actual read count}}{\left(\frac{\text{average gene length}}{1,000})(\frac{\text{sequencing depth}}{10^{6}}\right)}$.

### Data availability.

All amplicon sequencing data generated in this study can be accessed on the US National Center for Biotechnology Information SRA database under BioProject PRJNA587309. All gas and SCFA measurements, ASV tables, and code for data analysis are available at https://github.com/cusoiv.
