# Supplementary figures and images for: Prebiotics and Community Composition Influence Gas Production of the Human Gut Microbiota
Source: mBio. 2020 Sep 8;11(5):e00217-20. doi: 10.1128/mBio.00217-20 (PMC7482059; doi:10.1128/mBio.00217-20)

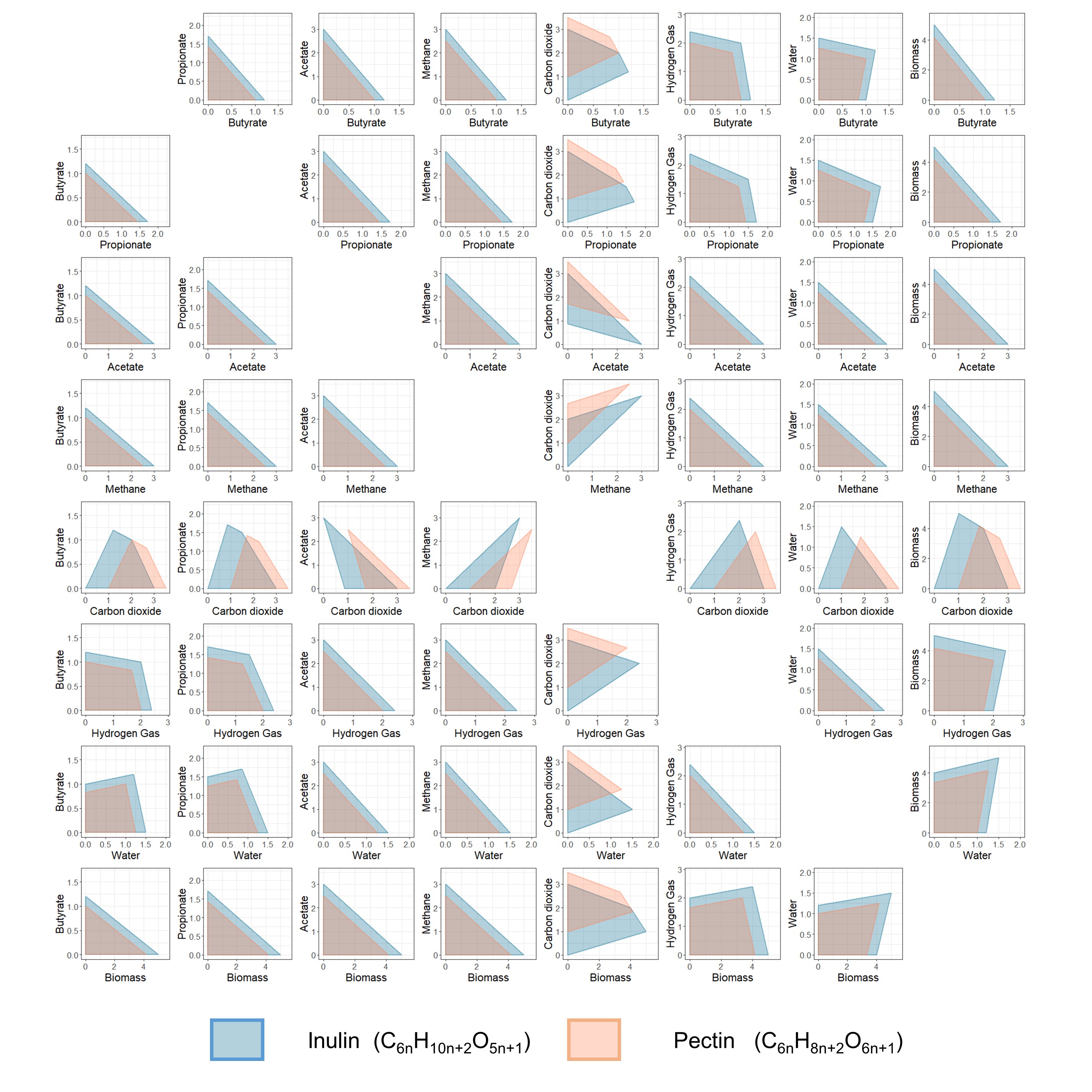

Supplement: FIG S1 [file mBio.00217-20-sf001.jpg]

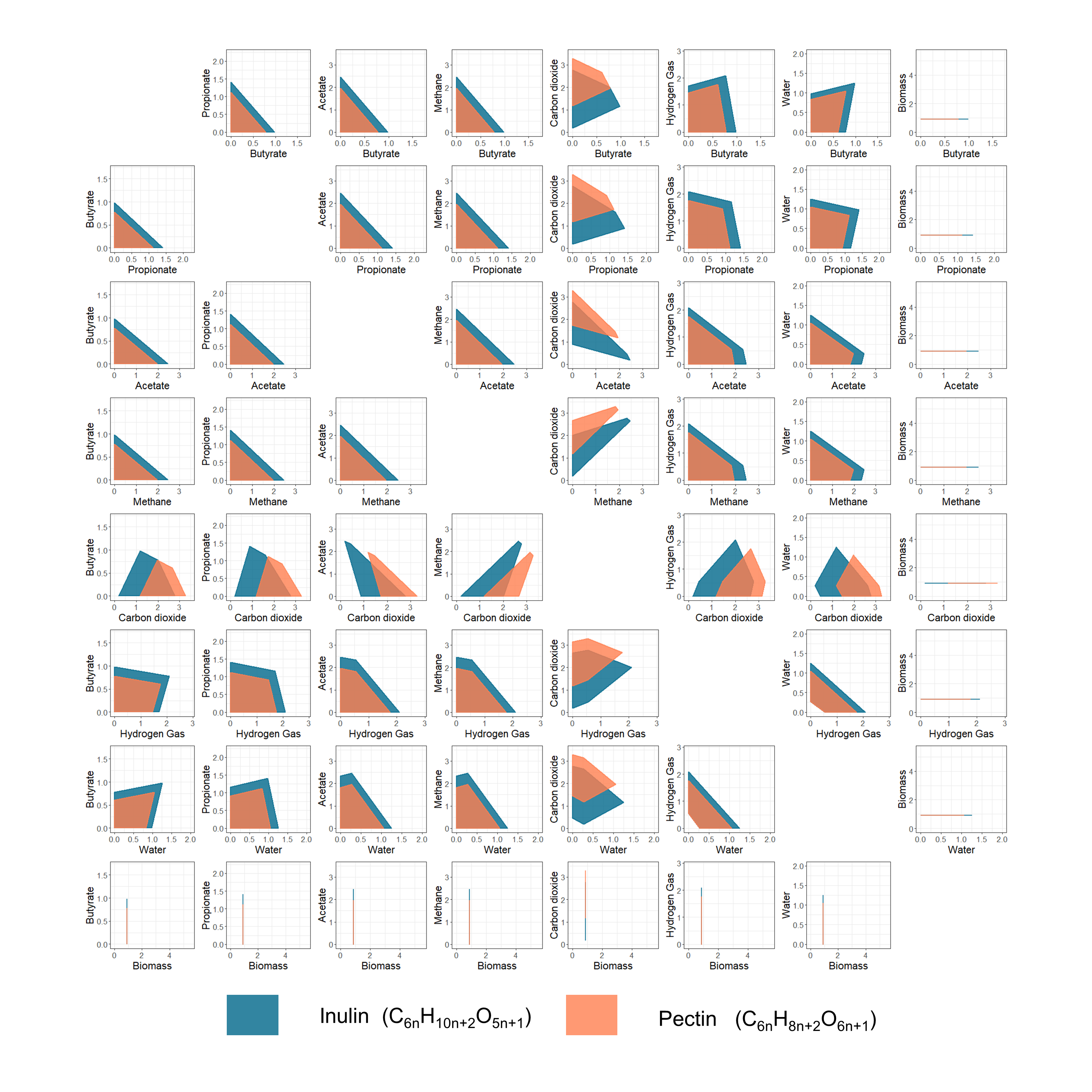

Supplement: FIG S2a [file mBio.00217-20-sf02a.tif]

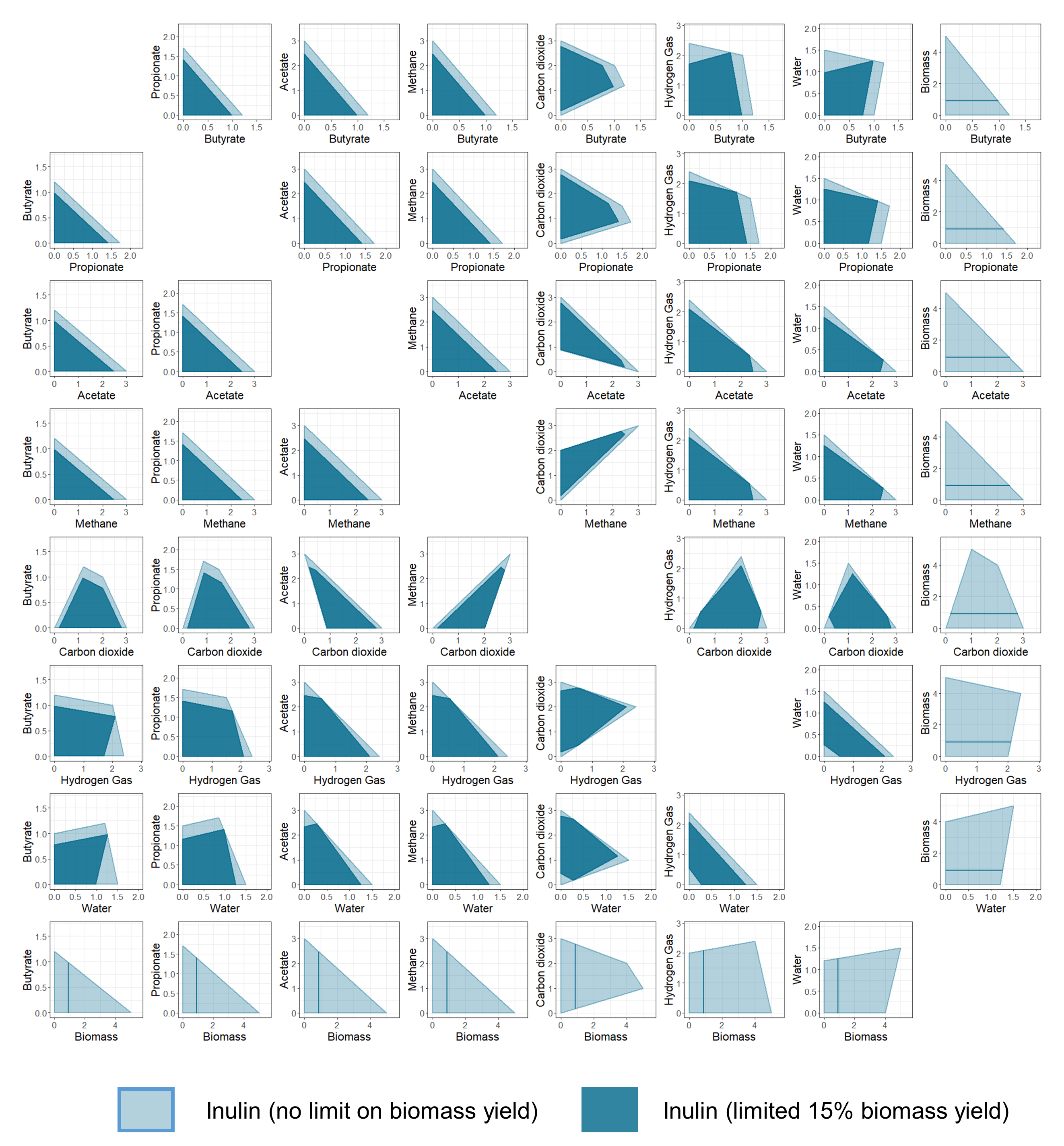

Supplement: FIG S2b [file mBio.00217-20-sf02b.tif]

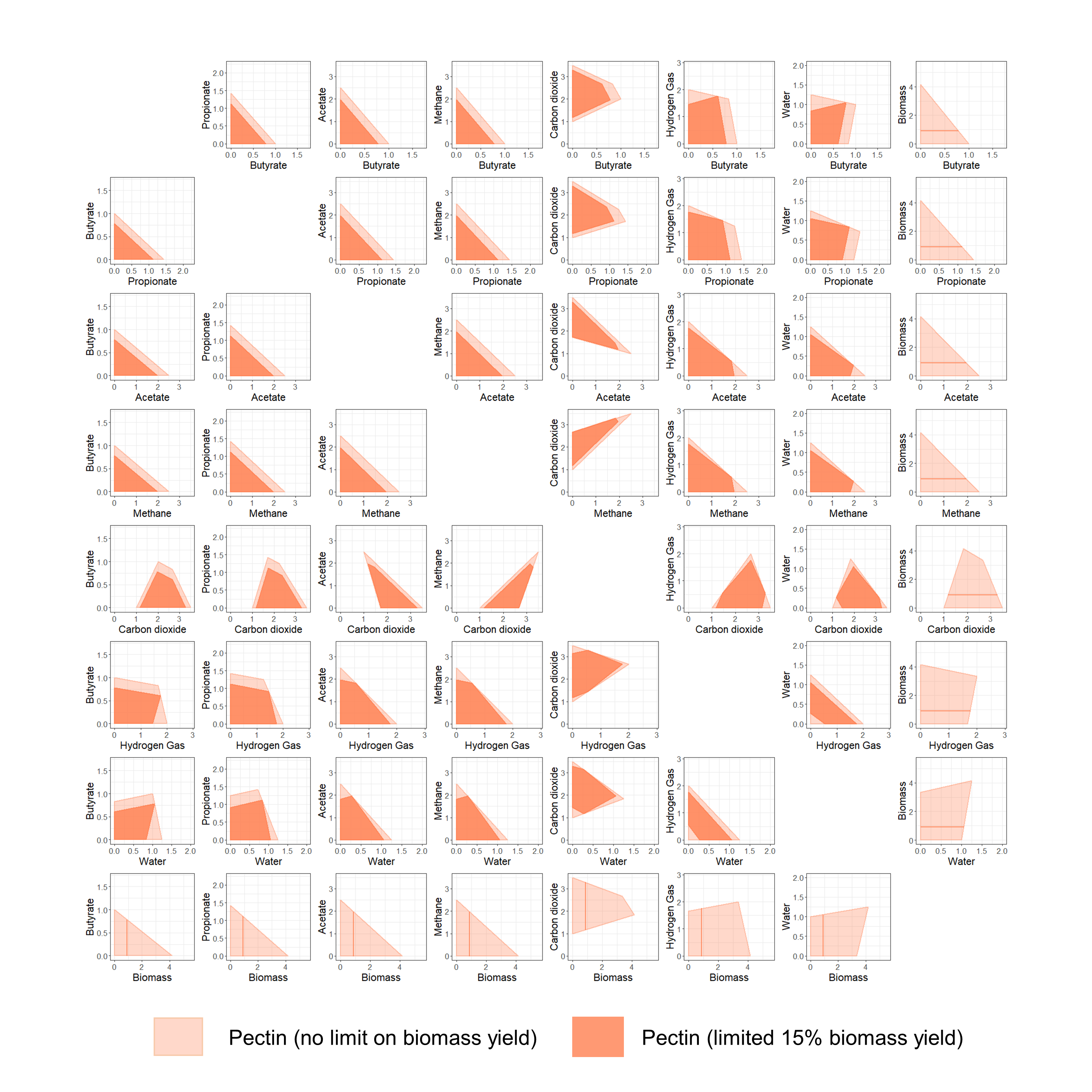

Supplement: FIG S2c [file mBio.00217-20-sf02c.tif]

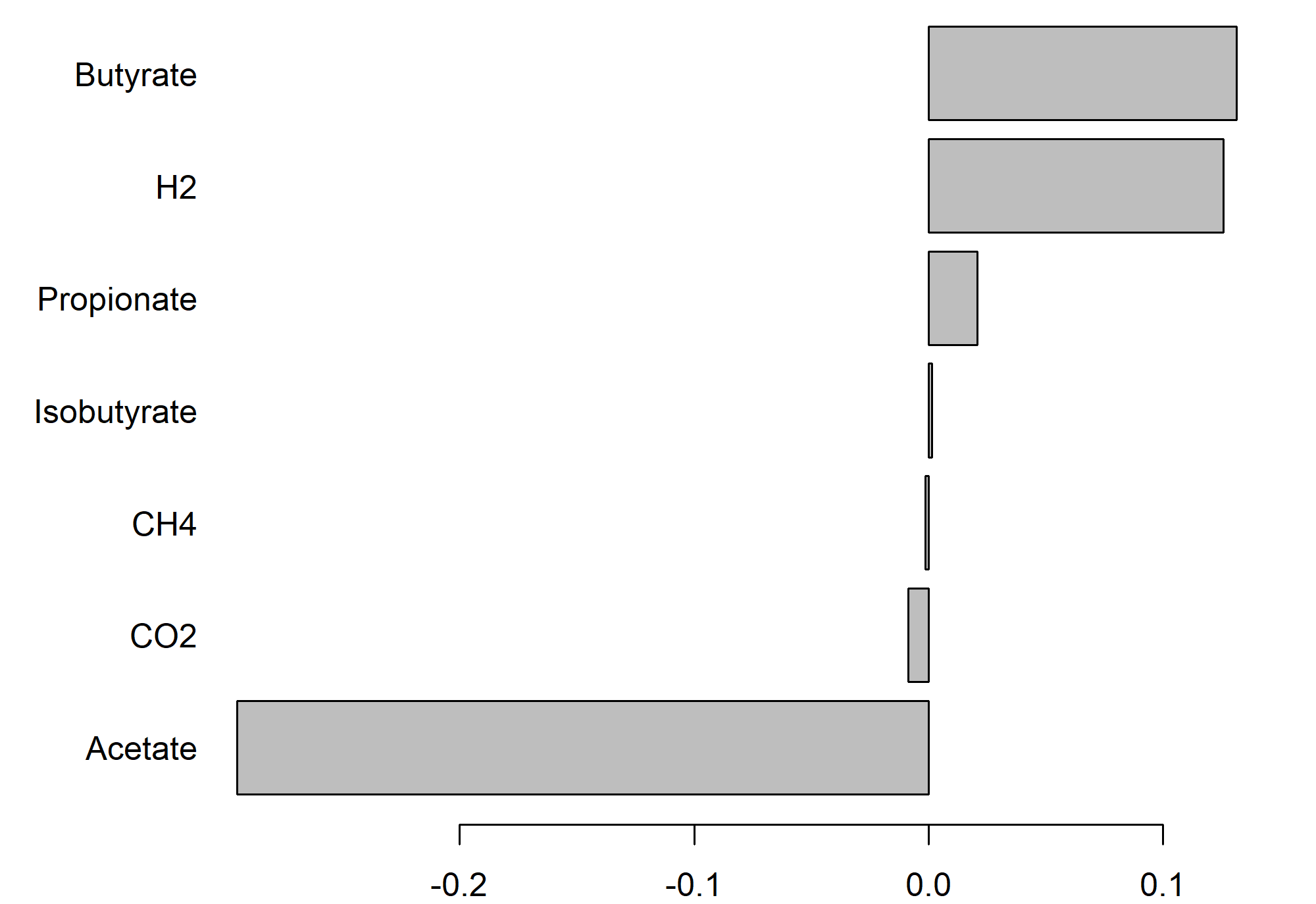

Supplement: FIG S3 [file mBio.00217-20-sf003.tif]

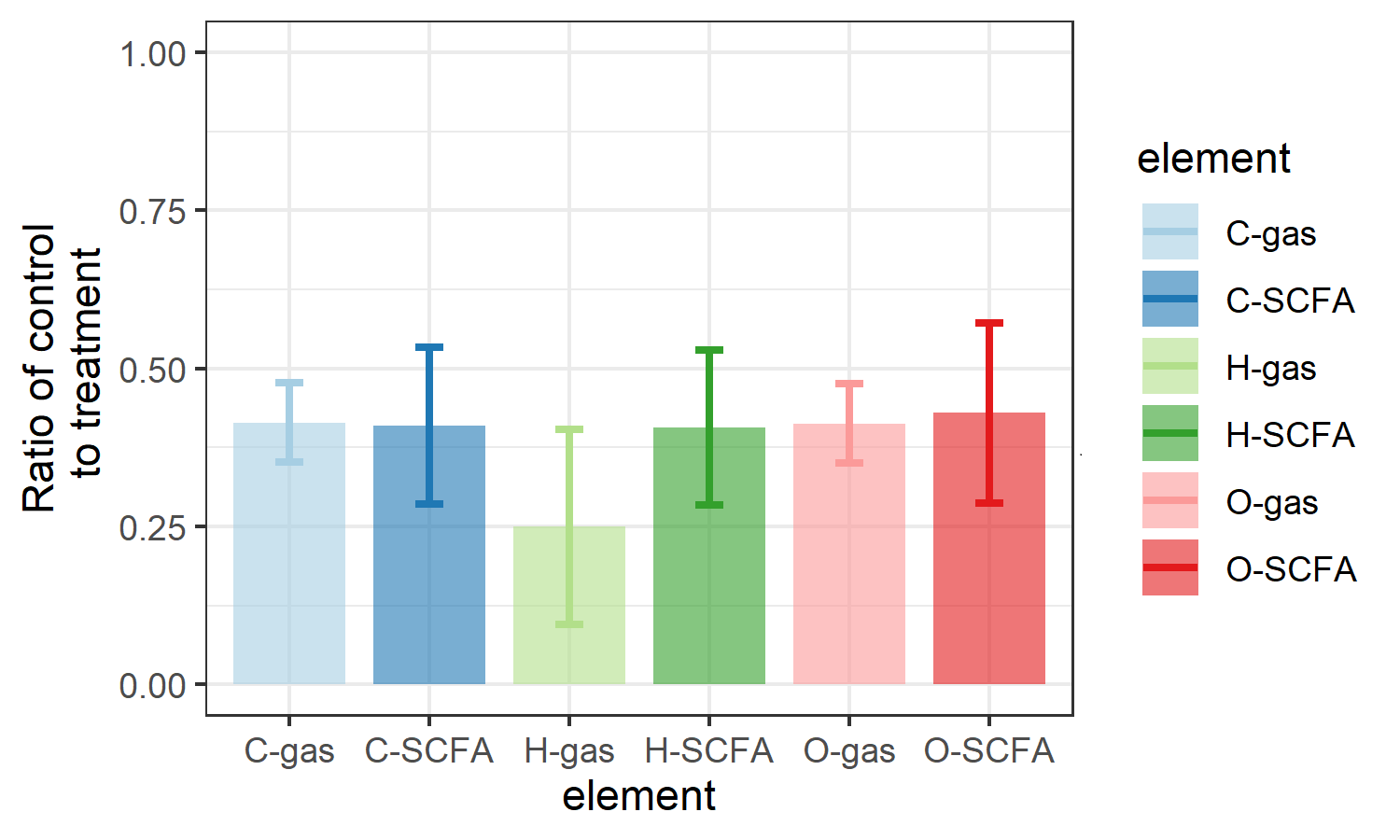

Supplement: FIG S4 [file mBio.00217-20-sf004.tif]

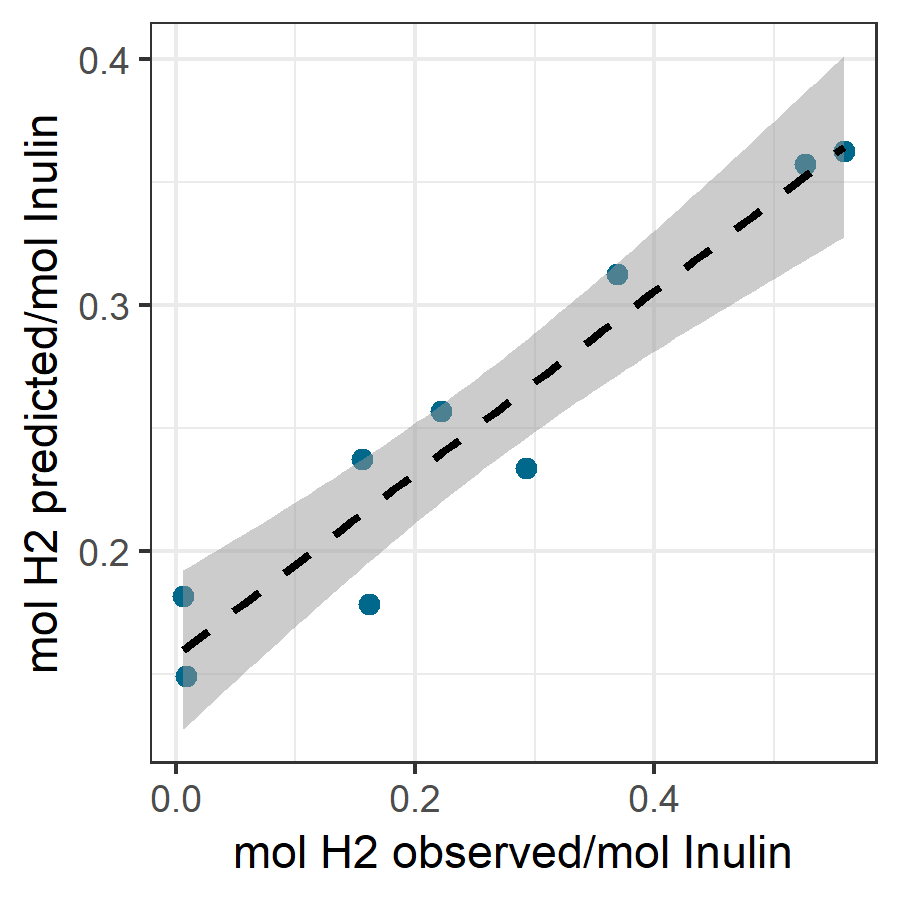

Supplement: FIG S5 [file mBio.00217-20-sf005.tif]

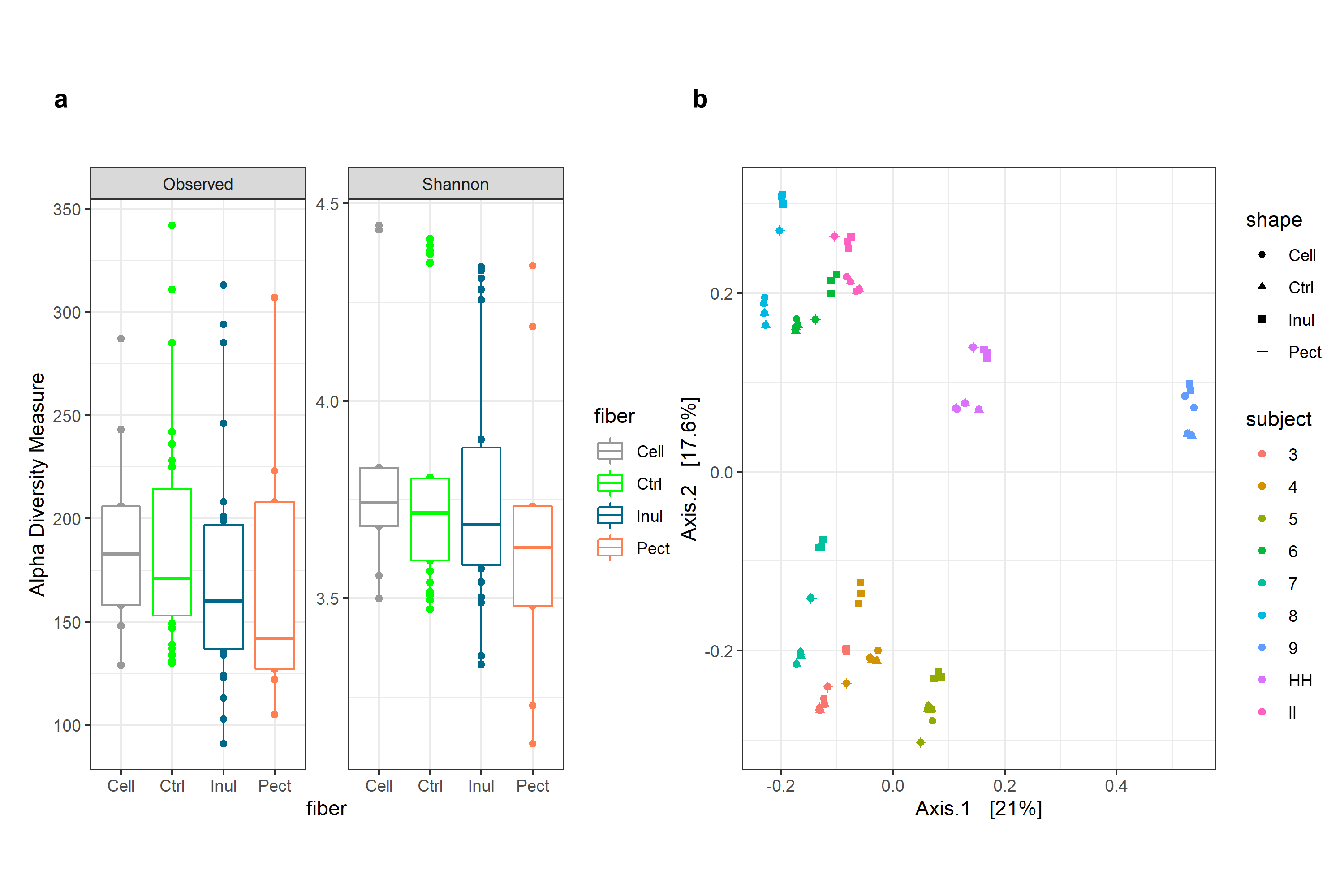

Supplement: FIG S6 [file mBio.00217-20-sf006.tif]
