# Supplementary material for: Prebiotics and Community Composition Influence Gas Production of the Human Gut Microbiota
Source: mBio. 2020 Sep 8;11(5):e00217-20. doi: 10.1128/mBio.00217-20 (PMC7482059; doi:10.1128/mBio.00217-20)
Supplement: TEXT S1 [file mBio.00217-20-s0001.docx]

**Supplementary Text**

**Estimation of Sulfate content in *ex vivo* system**

The content of sulfate in human feces is estimated to be 2.7µmol/g, independent of diet (1). Since each serum bottle in our ex vivo system contains approximately 0.4g of feces, the sulfate content in each bottle is 1.08µmol, which would take 4.32umol of H_2_ to undergo complete reduction to hydrogen sulfide. This is lower/approximately the same compared to the net amount of H_2_ left in the serum bottles at our time of measurement (32±22 µmol for inulin, 3.2±1.4 µmol for pectin), and given that gross production should be not less than net production, it is likely that the amount of sulfate the *ex vivo* system does not support elimination of H_2_ based on sulfate reduction alone.

1. Florin T, Neale G, Gibson GR, Christl SU, Cummings JH. 1991. Metabolism of dietary sulphate: absorption and excretion in humans. Gut 32:766–773.
